# Virtual simulation with AneuShape™ software for microcatheter shaping in intracranial aneurysm coiling: a validation study

**DOI:** 10.3389/fneur.2023.1095266

**Published:** 2023-04-26

**Authors:** Zeng-Bao Wu, Ying Zeng, Hua-Qiu Zhang, Kai Shu, Gao-Hui Li, Jian-Ping Xiang, Ting Lei, Ming-Xin Zhu

**Affiliations:** ^1^Department of Neurosurgery, Tongji Hospital, Tongji Medical College, Huazhong University of Science and Technology, Wuhan, Hubei, China; ^2^ArteryFlow Technology Co., Ltd., Hangzhou, Zhejiang, China

**Keywords:** virtual simulation, microcatheter shaping, cerebral aneurysm, coil embolization, treatment outcome

## Abstract

**Background:**

The shaping of an accurate and stable microcatheter plays a vital role in the successful embolization of intracranial aneurysms. Our study aimed to investigate the application and the role of AneuShape™ software in microcatheter shaping for intracranial aneurysm embolization.

**Methods:**

From January 2021 to June 2022, 105 patients with single unruptured intracranial aneurysms were retrospectively analyzed with or without AneuShape™ software to assist in microcatheter shaping. The rates of microcatheter accessibility, accurate positioning, and stability for shaping were analyzed. During the operation, fluoroscopy duration, radiation dose, immediate postoperative angiography, and procedure-related complications were evaluated.

**Results:**

Compared to the manual group, aneurysm-coiling procedures involving the AneuShape™ software exhibited superior results. The use of the software resulted in a lower rate of reshaping microcatheters (21.82 vs. 44.00%, *p* = 0.015) and higher rates of accessibility (81.82 vs. 58.00%, *p* = 0.008), better positioning (85.45 vs. 64.00%, *p* = 0.011), and higher stability (83.64 vs. 62.00%, *p* = 0.012). The software group also required more coils for both small (<7 mm) and large (≥7 mm) aneurysms compared to the manual group (3.50 ± 0.19 vs. 2.78 ± 0.11, *p* = 0.008 and 8.22 ± 0.36 vs. 6.00 ± 1.00, *p* = 0.081, respectively). In addition, the software group achieved better complete or approximately complete aneurysm obliteration (87.27 vs. 66.00%, *p* = 0.010) and had a lower procedure-related complication rate (3.60 vs. 12.00%, *p* = 0.107). Without this software, the operation had a longer intervention duration (34.31 ± 6.51 vs. 23.87 ± 6.98 min, *p* < 0.001) and a higher radiation dose (750.50 ± 177.81 vs. 563.53 ± 195.46 mGy, *p* < 0.001).

**Conclusions:**

Software-based microcatheter shaping techniques can assist in the precise shaping of microcatheters, reduce operating time and radiation dose, improve embolization density, and facilitate more stable and efficient intracranial aneurysm embolization.

## 1. Introduction

The precise insertion and stabilization of a microcatheter within an aneurysmal sac are crucial for a successful interventional embolization procedure (1–3). Therefore, it is essential to properly shape the microcatheter to achieve optimal navigation and stability (4). Inappropriate microcatheter shaping can cause the tip to rebound from the aneurysmal sac prematurely, obstructing further packing of the coil. Microcatheter shaping is not an easy process for neurosurgeons, even though it is a routine technique for the interventional embolization of intracranial aneurysms. Currently, microcatheter shaping mainly relies on the individual experience and estimation of operators and the visualization of the path of the microcatheter in the aneurysmal cavity and the parent artery according to digital subtraction angiography (DSA) (5). However, for aneurysms located in tricky areas or with intricate anatomical morphology, even sophisticated neurosurgeons may need to reshape the microcatheter or make multiple attempts to achieve the right shape to access the aneurysm sac (6). Some new methods of microcatheter formation have been reported in the literature; however, there are different limitations (6–8). The AneuShape™ software is a real-time planning tool that provides the microcatheter shaping template before the operation and simulates the path of the shaped microcatheter in the aneurysm sac and the parent artery with high accuracy, assisting neuro-interventionalists with accurate microcatheter shaping. In this research, we investigated the safety, accuracy, and effectiveness of using AneuShape™ software for microcatheter shaping during the endovascular embolization of intracranial aneurysms and also whether they were superior to traditional manual shaping methods.

## 2. Methods

### 2.1. Study participants

This was a retrospective single-institution series that was authorized by the institutional medical ethics committee. From January 2021 to June 2022, 105 consecutive patients with 105 single unruptured intracranial aneurysms in the anterior cerebrovascular circulation underwent endovascular therapy in our center. Neuro-interventionalists with 3–5 years of interventional surgery experience were in charge of the entire procedure. Software-based microcatheter shaping was applied in 55 cases, while conventional manual microcatheter shaping was applied in 50 cases. Collected data included demographics, aneurysm characteristics, treatment duration, radiation dose, and complications. The rates of microcatheter reshaping and accessibility, accurate positioning, and stability for shaping were also analyzed. All interventional therapies utilized coiling embolization. Meanwhile, the size of the aneurysm and personal intraoperative conditions determined whether stent-assisted coiling embolization was necessary.

### 2.2. Inclusion criteria

The inclusion criteria were as follows: (1) the presence of unruptured aneurysms specifically located in the anterior cerebrovascular circulation, (2) the absence of any surgical-related contraindications, and (3) the patient's voluntary consent to use AneuShape™ software.

### 2.3. Exclusion criteria

The exclusion criteria for this study comprised the following parameters: (1) giant aneurysms, ruptured aneurysms, and aneurysms located in the posterior cerebrovascular circulation and (2) the use of double or multiple microcatheter coiling technology.

### 2.4. Endovascular procedure

Before the endovascular procedure, the related clinical data were obtained from the patient's medical records, and all patients provided written informed consent. General anesthesia was used in all patients. The femoral sheath was the site of catheter placement. To maintain an active clotting time of ≥250 s, the patients were given one dose of standard heparin (70–100 IU/kg), which was subsequently administered in additional hourly doses (1,000 IU). A 6 French Navien (Stryker, USA) or guiding catheter (Envoy; Johnson & Johnson Health Care Systems Inc., USA) was advanced to the petrous horizontal segment of the internal carotid artery. Afterward, the shaped microcatheter was inserted into the aneurysm sac through a guiding catheter or Navien with microguidewire guidance. Then, the aneurysm sac was densely embolized with coils. For cases requiring stent assistance, the endovascular procedure utilized the “coil-through” or “stent semi-jailing” technique.

### 2.5. Antiplatelet therapy protocol

Before the unruptured aneurysm was treated with a stent, dual antiplatelet treatment with aspirin (100 mg/d) and clopidogrel (75 mg/d) was administered for 3–5 days. In addition, tirofiban (Gland Pharma Limited, China) at 0.1 μg/kg of body mass/min was administered by continuous intravenous infusion pump for 12 h. After the embolization procedure, patients who received stent assistance were advised to maintain dual antiplatelet therapy daily for at least 3 months and 100 mg of aspirin for a minimum of 6 months.

### 2.6. Virtual simulation with AneuShape™ software for microcatheter shaping

**Figure 1:**
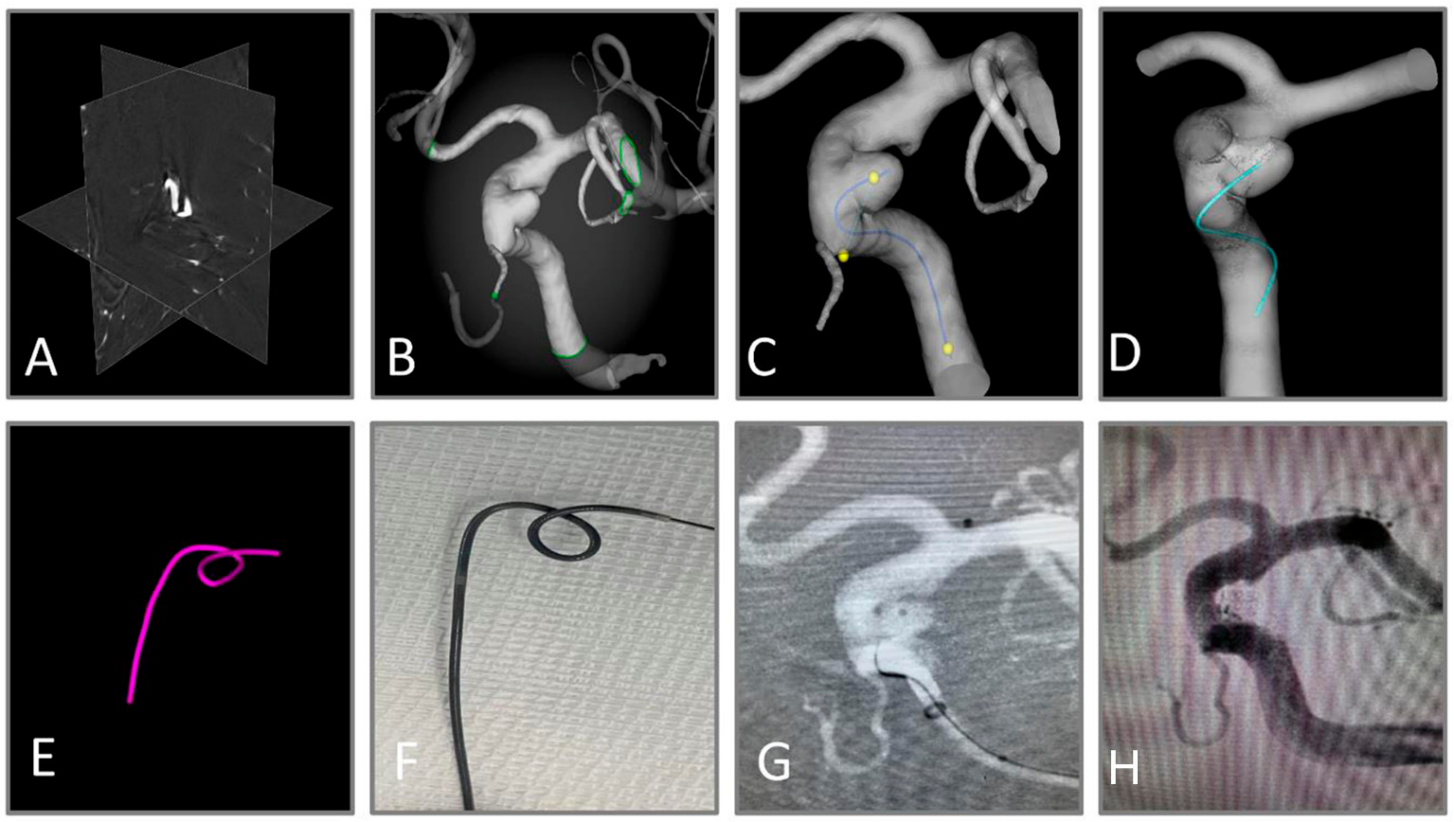
Workflow of virtual shaping of microcatheter with AneuShape™. **(A)** Reading of 3D RA images. **(B)** Segmentation and reconstruction of intracranial vessels. **(C)** Generation of the centerline, and the picking of three key points. **(D, E)** Visualization of anticipated microcatheter after removal of the mandrel (cyan) and that with mandrel inserted after shaping (purple). **(F)** The microcatheter was shaped manually using a template generated by the software. **(G)** The aneurysm sac was successfully catheterized through the shaped microcatheter. **(H)** Satisfactory aneurysm embolization.

AneuShape™ (ArteryFlow Technology, Hangzhou, China) is a real-time planning tool to assist neuro-interventionalists in microcatheter shaping. The workflow of AneuShape™ begins with reading the preoperative 3D rotational angiography (3DRA) images (Figure 1A). Based on level-set segmentation, the intracranial vasculature is then reconstructed, and a region of interest (ROI), for example, the left paraclinoid aneurysms of the internal carotid artery and parent artery, can be manually extracted with a cropping sphere (Figure 1B). To determine the direction of the virtual catheter, a centerline starting from the proximal inlet to the aneurysm sac should be generated (Figure 1C), which is accomplished by clicking at the proximal inlet and the aneurysm dome. Then, doctors need to pick two points on the centerline representing the proximal location and the tip of the anticipated microcatheter pathway, as well as a third point that corresponds to the first contacting point between the virtual microcatheter and the vessel wall along the distal-to-proximal direction from the tip (Figure 1C). The virtual microcatheter will then appear in cyan in only a few seconds (Figure 1D). If the surgeon defines the shaping factor (which describes the blunting behavior of the microcatheter after shaping, e.g., a 90°-shaping angle with an inserted mandrel turning to 45° after the removal of the mandrel if this factor equals 2.0) and a shaping length, a purple tube will appear in real-time, showing the shaped path of the microcatheter after inserting the mandrel (Figure 1E).

The principle of virtual microcatheter simulation includes a collision detection algorithm and a direction correction algorithm. At first, the virtual microcatheter “grows” from the tip location (usually at the centroid of the sac) and is picked up on the centerline toward the first contacting point. The “growing” process indicates that trial points are generated one by one along the direction defined by the previous two points. Meanwhile, collision detection is performed for each trial point, which is based on the widely used ray-casting algorithm. If the current trial point is outside the vessel wall, then its nearest neighbor on the centerline is located. The connecting line between the current trial point and its nearest neighbor intersects with the vessel wall, and the former is then moved to this intersecting location (and the “growing” direction is corrected correspondingly) to keep it inside the vessel wall. Then, a new trial point is generated along the new direction, and the aforementioned process repeats until the trial point reaches the proximal location picked on the centerline. Finally, all the points are connected sequentially. The virtual microcatheter is a set of adjacent line segments and is visualized as a winding tube in the vessel lumen.

To convert the microcatheter pathway in the vessel to its shaped pathway, we calculated the rotation matrix between each two adjacent line segments. The rotation matrix maps a vector rigidly to a new vector by taking a rotating axis and a rotating angle as inputs. The rotating axis between two line segments is the cross-product of their direction vectors. The rotating angle is obtained by multiplying the angle between the direction vectors of the two adjacent line segments with a coefficient (which equals the shaping factor minus one). Based on the rotation matrix, each line segment is rotated one by one with respect to its proximal neighbor until the desired shaping length is reached.

### 2.7. Manual microcatheter shaping

By utilizing a template generated by software, the microcatheter was manually molded and steamed for 60 s to complete the shaping (Figure 1F). The crawling path of the virtual microcatheter and the shaped path together facilitated the neuro-interventionalists in optimizing the surgical plan. The aneurysm sac was catheterized in one attempt by the shaped microcatheter (Figure 1G). The aneurysm was embolized with coils until satisfactory saccular obliteration was accomplished (Figure 1H).

### 2.8. Microcatheter evaluation and data collection

Two experienced neuro-interventionists determined whether it was “good” or “poor” in assessing the accessibility, the in-position condition, and the stability of each shaped microcatheter during procedures (9). Whether the microcatheters needed reshaping was also recorded. If the aneurysm sac was catheterized in 5 m, the accessibility of the microcatheter was defined as “good.” The position was defined as “poor” if the microcatheter tip was adherent within the intracranial aneurysm sac. However, if the microcatheter prematurely retreated out of the aneurysm sac and affected the further packing of the coil, stability was defined as “poor.” The fluoroscopy duration was measured in minutes and was defined as the time required from the beginning, when the microcatheter was out of the guiding catheter, to successful entry into the aneurysm sac and the time taken to release all coils. However, the time required to deploy the guiding catheter and stent was excluded. If there were multiple attempts at microcatheter delivery, the time for all attempts was recorded. The radiation dose was defined as the dose generated over the duration of treatment in terms of air kerma in mGy. We defined paraclinoid artery and posterior communicating artery aneurysms as proximal and middle cerebral artery bifurcations and anterior communicating artery aneurysms as distal aneurysms. The difference in efficiency between the distal and proximal aneurysms using software-assisted microcatheter shaping was also compared.

The aneurysm embolization rate and complications were also evaluated and noted by two independent neuro-interventionists. We applied the modified Roy-Raymond classification (MRRC) to assess the immediate angiographic results, and the classification criteria include (9): Class I (complete obliteration), Class II (neck residual), and Class III (non-complete occlusion). Our research designated Class I and Class II as successful intracranial aneurysm embolization.

### 2.9. Statistical analysis

Continuous data were expressed as the mean ± standard deviation (SD) and compared using the *t*-test, which conformed to a normal distribution, while the Mann–Whitney *U*-test was used for data that no longer fit the normal distribution. Categorical data were expressed as numbers (percentages) and compared using the chi-squared test. A *p*-value of < 0.05 was defined as statistically significant. The SPSS 22.0 software (IBM, USA) was used for statistical analysis.

## 3. Results

### 3.1. Patients

A total of 105 cases involved 50 patients who were not treated with software-assisted technology and 55 patients treated with software-based technology. Table 1 shows the baseline characteristics of cases treated with and without software-based technology. The mean age of patients was 56.71 ± 8.95 years. There were 71 (67.62%) sidewall aneurysms and 34 (32.38%) bifurcation aneurysms, which were all located in the anterior circulation. The mean maximum diameter, neck width, and dome/neck ratio of all aneurysms were 4.40 ± 2.30, 5.25 ± 1.99, and 0.79 ± 0.36 mm, respectively. Moreover, there were no statistical differences in morphological parameters between the two groups (*p* > 0.05; Table 1).

**Table 1:** Baseline clinical data and aneurysm characteristics in the software and manual groups.

**Factor**	**With software**	**Without software**	***p*-value**
	***n* = 55**	***n* = 50**	
Age, years mean ± SD	57.16 ± 9.30	56.22 ± 8.63	0.592
Male sex, *n* (%)	15 (27.27)	16 (30.0)	0.596
Dmax, mm mean ± SD	4.58 ± 2.64	4.21 ± 1.88	0.89
Neck, mm mean ± SD	5.23 ± 2.05	5.27 ± 1.95	0.90
**Size (%)**			0.194
Small (< 7 mm)	46 (83.60)	46 (92.00)	
Large (≥7 mm)	9 (16.40)	4 (8.00)	
AR	0.80 ± 0.35	0.78 ± 0.41	0.579
SR	1.57 ± 1.03	1.38 ± 0.88	0.49
**Sidewall/bifurcation aneurysm (%)**			0.937
Sidewall, *n* (%)	37 (67.27)	34 (68.0)	
Bifurcation, *n* (%)	18 (32.73)	16 (32.0)	
**Location (%)**			0.983
Paraclinoid aneurysms, *n* (%)	20 (36.30)	19 (38.00)	
PcomA aneurysms, *n* (%)	17 (30.90)	15 (30.00)	
AcomA aneurysms, *n* (%)	9 (16.40)	7 (14.00)	
MCA-Bifurcation aneurysms, *n* (%)	9 (16.40)	9 (18.00)	
**Treatment therapy (%)**			0.678
Coiling, *n* (%)	14 (25.50)	11 (22.00)	
Stent-assisted coiling, *n* (%)	41 (74.50)	39 (78.00)	

SD, standard deviation; AR, aspect ratio; SR, size ratio; PcomA, posterior communicating artery aneurysm; AcomA, anterior communicating artery aneurysm; MCA, middle cerebral artery.

### 3.2. Initial outcomes

In the software group, three aneurysms required an adjunctive procedure in which the microcatheter was discarded and a new one was reshaped for replacing the old one. However, in the group without software, 12 aneurysms required a manual procedure in which the microcatheter was discarded and a new one was shaped.

For both small (< 7 mm) and large (≥7 mm) aneurysms, the software group used more coils than the manual group (3.50 ± 0.19 vs. 2.78 ± 0.11, *p* = 0.008 and 8.22 ± 0.36 vs. 6.00 ± 1.00, *p* = 0.081, respectively; Table 2).

**Table 2:** Number of coils used.

	**Small aneurysms**<**7 mm**			**Large aneurysms** ≥**7 mm**		
	**With software**	**Without software**	***p*** **-value**	**With software**	**Without software**	***p*** **-value**
	***n*** = **46**	***n*** = **46**		***n*** = **9**	***n*** = **4**	
Dmax, mm mean ± SD	3.59 ± 0.20	3.86 ± 0.21	0.419	9.63 ± 0.55	8.22 ± 0.94	0.178
Coils (*n*)	3.50 ± 0.19	2.78 ± 0.11	0.008	8.22 ± 0.36	6.00 ± 1.00	0.081

In comparison with the manual group, the software group gained a shorter fluoroscopy duration (23.87 ± 6.98 vs. 34.31 ± 6.51 min, *p* < 0.001) and a lower radiation dose (563.53 ± 195.46 vs. 750.50 ± 177.81 mGy, *p* < 0.001) in terms of the univariate analysis (Table 3). In the coil-only group, the fluoroscopy duration and radiation dose were 16.88 ± 0.75 vs. 28.50 ± 1.16 min, *p* < 0.001, and 381.00 ± 18.83 vs. 597.27 ± 33.32 mGy, *p* < 0.001, respectively (Table 4). Software-based microcatheter shaping techniques also reduced operating time and radiation dose in the stent-assisted coiling group, which were 26.25 ± 0.99 vs. 35.95 ± 0.99 min, *p* < 0.001 and 625.85.00 ± 28.93 vs. 793.72 ± 27.19 mGy, *p* < 0.001, respectively (Table 4). Meanwhile, we found that there was a linear correlation between fluoroscopy duration and radiation dose (Spearman's correlation coefficient = 0.944, *p* < 0.001).

**Table 3:** Outcomes and assessment of microcatheter shaping.

	**With software**	**Without software**	***p*-value**
	***n* = 55**	***n* = 50**	
**Outcomes**			
Need for reshaping, *n* (%)	12 (21.82)	22 (44.00)	0.015
Duration of the intervention, mean ± SD (min)	23.87 ± 6.98	34.31 ± 6.51	< 0.001
Radiation dose, mean ± SD (mGy)	563.53 ± 195.46	750.50 ± 177.81	< 0.001
**Postoperative angiography**			
Raymond grade 1, *n* (%)	41 (74.54)	24 (48.00)	0.005
Raymond grade 2, *n* (%)	7 (12.73)	9 (18.00)	0.453
Raymond grade 3, *n* (%)	7 (12.73)	17 (34.00)	0.010
Complication, *n* (%)	2 (3.60)	6 (12.00)	0.107
**Microcatheter shaping**			
Accessibility, good, *n* (%)	45 (81.82)	29 (58.00)	0.008
Positioning, good, *n* (%)	47 (85.45)	32 (64.00)	0.011
Stability, good, *n* (%)	46 (83.64)	31 (62.00)	0.012

**Table 4:** Software-based microcatheter shaping techniques reduce operating time and radiation dose both in coiling-only group and stent-assisted coiling group.

	**Coiling**			**Stent-assisted coiling**		
	**With software**	**Without software**	***p*** **-value**	**With software**	**Without software**	***p*** **-value**
	***n*** = **14**	***n*** = **11**		***n*** = **41**	***n*** = **39**	
Dmax, mm mean ± SD	2.20 ± 0.12	2.25 ± 0.14	0.766	5.39 ± 0.40	4.76 ± 0.28	0.45
Duration of the intervention, mean ± SD (min)	16.88 ± 0.75	28.50 ± 1.16	< 0.001	26.25 ± 0.99	35.95 ± 0.99	< 0.001
Radiation dose, mean ± SD (mGy)	381.00 ± 18.83	597.27 ± 33.32	< 0.001	625.85.00 ± 28.93	793.72 ± 27.19	< 0.001

In addition, the software group gained a lower reshaping rate for microcatheters (21.82 vs. 44.00%, *p* = 0.015) and a higher rate of accessibility (81.82 vs. 58.00%, *p* = 0.008) and achieved a better positioning (85.45 vs. 64.00%, *p* = 0.011) and higher stability (83.64 vs. 62.00%, *p* = 0.012; Table 3). Compared with the proximal aneurysms, the efficiency of using software-assisted microcatheter shaping was not reduced, and the difference between the two groups was not statistically significant (Table 5).

**Table 5:** Comparison of microcatheter shaping efficiency of proximal and distal aneurysms by software-assisted.

**With software**	**Proximal aneurysms**	**Distal aneurysms**	***p*-value**
	***n* = 37**	***n* = 18**	
Duration of the intervention, mean ± SD (min)	23.18 ± 6.39	25.28 ± 8.08	0.300
Radiation dose, mean ± SD (mGy)	552.70 ± 190.60	585.78 ± 209.00	0.531
Accessibility, good, *n* (%)	31 (83.78)	14 (77.78)	0.866
Positioning, good, *n* (%)	32 (86.49)	15 (83.33)	1
Stability, good, *n* (%)	31 (83.78)	15 (83.33)	1

Post-embolism angiograms showed that the software group gained a higher rate of complete occlusion (Raymond-Roy Grade Scale I [RRGS I]: 74.54 vs. 48.00%, *p* = 0.005), a lower rate of neck remnant (RRGS II: 12.73 vs. 18.00%, *p* = 0.453), and non-complete occlusion (RRGS III: 12.73 vs. 34.00%, *p* = 0.010; Table 3), as well as a greater degree of complete or approximately complete aneurysm occlusion (87.27 vs. 66.00%, *p* = 0.010).

### 3.3. Perioperative complications

In the manual group, six patients (12.00%) experienced various complications: three minor strokes with mild motor symptoms degenerating, one small frontal hematoma with a mild headache, one asymptomatic internal carotid artery dissection, and one regional obliteration of the right middle cerebral artery with partly resolved hemiparesis. In the group with software, two patients (3.60%) experienced complications: one patient had a mild ischemic stroke with transient muscle weakness of the lower limbs, and another had a stroke with mild right hemiparesis and dysarthria. All complications except one (the middle cerebral artery partial occlusion in the manual group that caused permanent weakness of the upper limbs) completely reverted without sequelae.

## 4. Discussion

The optimal microcatheter shape has a significant impact on retaining the accuracy and stability of the microcatheter during embolization, enabling safe, and effective treatment of intracranial aneurysms (1, 2). In our series, the manual and software groups were compared. Microcatheter shaping based on the AneuShape™ software had reliable stability and accuracy in endovascular treatment and played a significant role in the successful embolization of intracranial aneurysms. Besides, the use of the real-time preoperative virtual shaping technique was associated with a significant reduction in the intervention duration and the radiation dose in both the coiling-only group and the stent-assisted coiling group.

Currently, most neuro-interventionalists use 3D-DSA images to evaluate the anatomy of aneurysms and their correlation to the parent artery before manually shaping the microcatheter. While microcatheter shaping is an essential technique for interventional treatment, it is sometimes difficult to obtain a satisfactory shape. One reason is that 3D imaging technology lacks depth information; hence, neuro-interventionalists cannot accurately perceive the 3D cerebrovascular morphology and the spatial position of microcatheters in that parent artery (4, 6). Second, it is hard for neuro-interventionalists to accurately identify the real route of the microcatheter into the parent artery, making them rely solely on their imagination. According to the characteristics of the microcatheter, neuro-interventionalists can adjust it into different shapes, but exaggerating the mandrel shape during the operation still depends on personal experience (6, 10). However, even experienced surgeons cannot guarantee that the shaping of the microcatheter is always appropriate, and sometimes reshaping is needed.

Furthermore, a recent multicenter study has shown that interventionists often use animal models for simulation training, and there is a lack of standardized training in neurological interventions (11). Hence, there is a need to develop a new artificial method that meets the training requirements of alternative animal models, which can increase the experience of neuro-interventionists faster. Software-based technology provides a promising method for interventional surgery simulation training and direct utilization of software-based technology during surgery.

Unlike conventional manual shaping processes that rely primarily on personal experience, the software can input DICOM data directly from 3D-DSA images during operation to generate microcatheter shaping templates. This makes it more accessible and convenient for doctors to mold the microcatheter at once and avoids repeated manual shaping, especially for less experienced neurosurgeons. In addition, software-based technology also offers a standardized manual training system for beginners, allowing neurosurgeons to quickly obtain intuitive experience and skills (12). In related research (12, 13), some researchers have proposed that the use of software-based technology to help surgeons shape the mandrel in the process of embolization of intracranial aneurysms achieves good clinical results. Liu et al. (12) reported that during endovascular surgery, the software-based simulation template entered the aneurysm sacs accurately and without any complications. Software-based microcatheter shaping was stable and accurate. However, these studies lacked a control group to assess the technical and clinical outcomes of software-based technology vs. traditional manual microcatheter shaping.

In our study, software-based microcatheter shaping was more accurate and stable compared with the manual shaping process. Therefore, we observed a higher rate of accessibility, achieving a better position and higher stability. In addition, the use of software-based technology reduced the need for reshaping the microcatheter or deploying a second one because of fatigue caused by repeated use. These findings were seldom quantified and reported in past research. These factors not only resulted in longer operating times and unproductive costs but also posed potential risks for procedural complications.

The reduction in duration with the software-based technology is likely related to the precise shaping of the microcatheter. As for complicated structures, neuro-interventionalists should perhaps remodel the microcatheter and make multiple attempts during the procedure to adapt the structural characteristics of the aneurysm sac and the parent artery. These procedures can prolong the procedural duration and raise the risk of aneurysm rupture and ischemic events (14–16). Software-based technology can reduce the need for repeated and inefficient maneuvers, making the procedure smoother and more efficient. Surgeons can obtain the template for microcatheter shaping with pivotal data, such as the angle and length of the microcatheter tip, in ~10 min. In our research, the operating time of the software-based group was reduced by about 30.43% compared to the manual group. Despite the reduced duration of the intervention, we found an increase in the number of coils used with the help of software-assisted shaping, implying denser aneurysm embolisms and lower recurrence rates. Our research results also showed that the software-based group achieved more complete or approximately complete aneurysm occlusions.

Longer operative times may be related to higher ischemic stroke rates during endovascular therapy (14, 15) or diagnostic angiography (16). It has been reported that the intervention treatment over 100–120 min was significantly associated with the risk of ischemic stroke events (14, 15). Similar results were shown in our study. Compared with the manual group, the complication rate in the software-based technology group was lower. Although the difference in the complication rate between those two groups was not significant, it appears plausible that a shorter operation time might reduce the risk of silent or symptomatic ischemic events.

Another focus of our research was the reduction in radiation dose, which is linearly related to the duration of fluoroscopy. Appropriate software-based microcatheter shaping reduces procedure time and, thus, facilitates a lower radiation dose. This is beneficial in reducing radiation-related risks for both patients and neuro-interventionalists.

This study has several limitations. First, this study used a retrospective design, which may have cause an introduced bias. Second, the shaping of the microcatheter by the surgeon according to the software-based template could still be subject to personal biases. Third, the number of cases was not large enough, and the data represented the limited experience of our institution. Fourth, due to the absence of randomization between the groups, the uniform distribution of aneurysms could not be ensured. Five, the impact of software-based techniques on clinical outcomes could not be better compared due to the lack of long-term follow-up. Six, software-based technology can improve the success rate of microcatheter shaping for low- and mid-level neuro-interventionists with 3–5 years of experience, but it is not clear whether it will assist the success rate of senior doctors (more than 5 years of experience). Therefore, further research is needed to investigate the use of the software by senior neuro-interventionists. Finally, multicenter randomized controlled trials are required to verify the effectiveness of this technology in the future.

## 5. Conclusions

In our experience, software-based technology is a beneficial tool that can assist neuro-interventionists in accurately shaping microcatheters. The use of this software improved embolization density, increased the rate of successful aneurysmal occlusion, and reduced the need for rectification procedures, fluoroscopy time, and radiation dose.

## Data availability statement

The raw data supporting the conclusions of this article will be made available by the authors, without undue reservation.

## Ethics statement

The studies involving human participants were reviewed and approved by the Institutional Medical Ethics Committee of Tongji Hospital. The patients/participants provided their written informed consent to participate in this study. Written informed consent was obtained from the individual(s) for the publication of any potentially identifiable images or data included in this article.

## Author contributions

Z-BW, TL, and M-XZ designed and conducted the experiments and statistical analysis and drafted the manuscript. YZ participated in the operation and the collection of clinical data. G-HL and J-PX were responsible for software processing and data curation. H-QZ and KS conducted intraoperative and postoperative evaluations. All authors contributed to the article and approved the submitted version.
